# Redox-responsive hyaluronan-conjugated polypyrrole nanoparticles targeting chemo-photothermal therapy for breast cancer

**DOI:** 10.3389/fbioe.2022.1049437

**Published:** 2022-10-24

**Authors:** Jingjun Sun, Shuangjiu Zhu, Weixuan Xu, Guoqin Jiang

**Affiliations:** ^1^ Department of Surgery, The Second Affiliated Hospital of Soochow University, Suzhou, China; ^2^ Department of Breast Surgery, Affiliated Maternity and Child Health Care Hospital of Nantong University, Nantong, Jiangsu, China; ^3^ Department of Thyroid and Breast Surgery, The Second People’s Hospital of Lianyungang City, Lianyungang, China; ^4^ School of Medicine, Nantong University, Nantong, Jiangsu, China

**Keywords:** polypyrrole, redox responsive, hyaluronan, photothermal effects, chemophotothermal therapy

## Abstract

The combination of chemo-photothermal therapy has a wide application prospect in the intensive treatment of cancer. In this study, we developed a complex nanoparticle consist of polypyrrole, cystine dihydrochloride and hyaluronan. The polypyrrole nanoparticles loaded with paclitaxel exhibited good photothermal effects, and the drug release can be triggered by combined response of temperature and redox. *In vitro* biological studies indicated the nanoparticles could effectively induced apoptosis of MDA-MB-231 breast cancer cells involved in the potential mechanism of inhibition of biological expression of heat shock proteins and JAK-STAT signaling pathway. In addition, the nanoparticles have a significant inhibitory effect on cancer growth in breast tumor-bearing mice model, indicating that they have great potential for synergistic chemo-photothermal therapy.

## Introduction

Breast cancer has the highest mortality and morbidity among women and poses a serious threat to women’s health (Bray et al., 2018). Cancer cells can be effectively killed by photothermal therapy through the thermal effect of photothermal agents under near-infrared irradiation (NIR) (de Melo-Diogo et al., 2017; Liu et al., 2019; Li and Pu, 2020; Luo et al., 2021; Zhang et al., 2021). Because NIR-II laser (1,000–1,350 nm) can penetrate tissue deeper and have better biocompatibility than NIR-I lights (650–950 nm), NIR-II-responsive photothermal agents have recently received much attention (Geng et al., 2020; Yu et al., 2021; Xu et al., 2022). Polypyrrole (PPy) is one of the commonly reported NIR-II-responsive photothermal agents, and it shows many advantages, such as facile preparation, good biocompatibility, and high photothermal conversion efficiency (Song et al., 2015; Mi et al., 2017; Guo et al., 2019; Xu et al., 2022). However, the researches on PPy-based nanoparticles with distinct absorbance in the NIR-II bio-window are limited.

The stimulatory-responsive drug delivery system has gained a lot of attention, which can promote the release of intracellular drugs and further enhance the killing effect of tumor. With the further understanding of cancer and the microenvironment of cancer tissue, scientists have confirmed that the proliferation, invasion and metastasis of cancer cell are highly related to the abnormal changes of cancer tissue microenvironment (Zhang et al., 2019). Through exploring the features of cancer tissue microenvironment, drug delivery system can be correspondingly designed to release drugs at specific sites or respond to specific stimuli, which will improve the delivery efficiency, enhance the bioavailability of drugs, as well as reduce toxic and side effects (Hossen et al., 2019). Glutathione (GSH) is a powerful antioxidant. There is higher concentration of GSH in cancer tissues than that in normal tissues. Therefore, redox properties can serve as potential stimuli to control drug release. It has been reported that the overexpression of CD44 receptor of hyaluronan (HA) existing in many different types of tumor cells (Misra et al., 2011). Therefore, HA is a potential tool to enhance the tumor targeting effect of drug carriers.

Herein, we aim to design a multi-functional PPy based nanoparticles that can be used in synergistic chemo-photothermal therapy. The multifunctional PPy based nanoparticles (PCH) consists of PPy, cystine dihydrochloride (Cys) and HA. Cys connecting arm can endow the nanoparticles with the characteristics of reduction response. Thus, this system can not only ablate tumors through photothermal effect, but also trigger drug release in tumor microenvironment by reduction-responsive stimuli. Structures, photothermal effect, and redox/temperature dependent drug release pattern of the PPy based complex nanoparticles were studied. In addition, cytostatic experiments and mechanisms were performed on MDA-MB-231 cells. The anti-tumor effects of paclitaxel (PTX) loaded PCH nanoparticles breast tumor-bearing mice were also evaluated.

## Materials and methods

### Materials

Pyrrole (Py) was obtained from Rhawn (Shanghai, China). Pyrrole-1-propionic acid (Py-COOH), polyvinyl alcohol (PVA, Mw: 89,000–98,000), 1-ethyl-3-(3-dimethylaminopropyl) carbodiimide hydrochloride (EDC), N-hydroxysuccinimide (NHS) and Ferric chloride hexahydrate (FeCl_3_) were purchased from Macklin (Shanghai, China). Hyaluronic acid [HA, MDMW: 22 × 105 Da)] was supplied by Nantong Feiyu Biotechnology Co., Ltd., Paclitaxel (PTX) was obtained from Yangtze Pharmaceutical Group Co., Ltd., Cystine dihydrochloride (Cys) from Shanghai Aladdin Biotechnology Co., Ltd.

### Synthesis and characterization of nanoparticles

PPy-COOH nanoparticles were firstly prepared according to previous work. Briefly, FeCl_3_ (0.6 g) was added to 10 ml 8% PVA solution and stirred for 1 h. Then, Py and Py-COOH (0.376 g) was added into the solution at 4°C, the mass ratio of Py to Py-COOH was set as 1:1. The chemical oxidation polymerization of Py/Py-COOH monomer was carried out for 4 h at 4°C. 0.24 mg EDC and 0.192 mg NHS were dissolved in 20 ml water, and then added into the PPy-COOH nanoparticles solution for 2 h. After that, 0.6 g Cys was added into above activated PPy-COOH nanoparticles solution and stirred for 24 h at room temperature. PPy-Cys nanoparticles were obtained through centrifugation and purification. 12 mg EDC and 9 mg NHS was added in to 2 ml HA solution for 0.5 h. Then, it was added into 18 ml above PPy-Cys nanoparticles solution and stirred for 12 h at room temperature. Finally, PPy-Cys-HA (PCH) nanoparticles were collected through centrifugation and purification.

Chemical analysis of the nanoparticles was characterized using a FTIR (TENSOR 27, Bruker, Germany). Samples were scanned at resolution of 2 cm^−1^ in transmission mode. Scanning range is from 4,000 to 400 cm^−1^. Morphological information of the nanoparticles was collected using a scanning electron microscopy (SEM, ZEISS Gemini SEM 300, Germany). Diameter of the nanoparticles were measured using an ImageJ 1.40 G software.

### Near-infrared irradiation-photothermal effect

Aqueous solutions of PCH nanoparticles at different concentrations (0, 0.5, and 1 mg/ml) were irradiated with a 1,064 nm laser (1.0 W/cm^2^) for 5 min. Photothermal images were caught every 1 min using a thermal imaging camera (FLIR C3, Fluke, United States).

### Drug loading and release

PTX-loaded PCH (PTX/PCH) nanoparticles were also prepared as described above. The *in vitro* release of PTX (initial concentration: 50 μg/ml) from PTX/PCH nanoparticles (0.5 ml) was investigated using a dialysis bag at 37°C in 25 ml PBS (10 mM, pH 7.4) either in the presence or absence of 20 mM DTT (to simulate a highly reduced tumor microenvironment). At predetermined time intervals, 5 ml of release buffer was sucked out and supplemented with an equal volume of fresh buffer. The amount of released PTX was measured by high performance liquid chromatography (HPLC).

### Cytotoxicity study

MDA-MB-231 cells were seeded into 96-well plates (8 × 10^3^ cells/well) and incubated with 0.5 mg/ml PCH and PTX/PCH nanoparticles for 24 h. Cells treated with PBS were served as control. In addition, MDA-MB-231 cells were laser-irradiated with PCH and PTX/PCH nanoparticle groups for 5 min and further incubated for 24 h. CCK-8 assay was used to determine the viability. Cells were stained with dead/live kit for 15 min. Then, cells were washed with PBS and observed by fluorescence microscope (Nikon Ti-DH).

### Cell apoptosis assay

MDA-MB-231 cells were seeded into 12-well plates (5 × 10^5^ cell/ml) and incubated for 24 h. Cells were washed twice with PBS and medium containing 0.5 mg/ml PCH and PTX/PCH nanoparticles were added, respectively. After being cultured for 12 h, the cells were washed twice with PBS and 1 ml fresh medium was added. NIR groups were irradiated with a 1,064 nm laser at a power of 1 W/cm^2^ for 1 min and non-NIR groups were not treated. After incubation of another 6 h, the cells were washed twice with PBS and digested with trypsin. The cells were centrifuged and collected. Finally, the cells were processed according to the instructions in the MuseTM Annexin V and Dead Cell Kit (Merck, Germany). The result was recorded using Muse Cell Analyer (Merck, Germany).

### Intracellular reactive oxygen species detection

To detect the amount of ROS, MDA-MB-231 cells were incubated with different samples and then the probe DCFH-DA (10 μM) was added to each well. 30 min later, the medium was removed, and the cells were washed with PBS and observed under a fluorescence microscope (Nikon Ti-DH).

### Western blot analysis

MDA-MB-231 cells were seeded on a 6-well plate and cultured for 12 h. After different treatments, cells were harvested and washed three times with PBS. The cells were then collected and further lysed for Western blot analysis.

### High-throughput RNA-seq

For transcriptomic sequencing, MDA-MB-231 were seeded onto a 6-well plate and cultured overnight. After distinct treatments, the cells were harvested, and TRIzol was used for total RNA isolation. The isolated total RNA was stored in liquid nitrogen ready for use. RNA concentration was determined and total RNA quality inspection was performed. RNA libraries were then prepared by PCR amplification. Finally, sequencing was performed on the RNA-seq platform according to the manufacturer’s instruction, and the data obtained were processed by Genewiz Co. Ltd.

### *In-vivo* antitumor efficacy

Therapeutic effect of PTX-loaded PCH nanoparticles was evaluated in nude mice bearing MDA-MB-231 breast cancer xenografts. All the animal experimental procedures were approved by the ethical committee of Soochow University. When the volume of the tumor was in the range of 30–40 mm^3^, the treatments were initiated (day 0). The mice were randomly divided into two groups, and there are six in each group. All mice were injected with PTX/PCH nanoparticles (5 mg PTX equiv./kg) *via* tail vein. The samples were continuously irradiated with a 1,064 nm laser (1 W/cm^2^) for 5 min. The temperature elevation and photothermal images of mice were recorded by using a thermal imaging camera (FLIR C3, Fluke, United States). The treatment was repeated every 3 days for 5 times. The tumor volume was calculated according to the formula V = 0.5 × L × W × H, where L, W, and H are the tumor dimensions at the length, width and height, respectively. At the end of the treatment, the tumor, liver, heart and kidney tissues were removed from mice. The tissues were fixed with 4% paraformaldehyde for 24 h and dehydrated with 30% sucrose solution for 48 h. The tissues were frozen at −80°C and sectioned with 8 μm thickness. The sliced organ tissues were stained by hematoxylin and eosin (H&E) and observed.

### Statistical analysis

All data are presented as the mean ± standard deviation (SD). One-way analysis of variance (ANOVA) of Origin 8.0 was used to determine significance among groups. When *p* value was less than 0.5, the difference was significant. *p < 0.05, **p < 0.01, ***p < 0.001.

## Results and discussion

### Synthesis and characterization of nanoparticles

**FIGURE 1:**
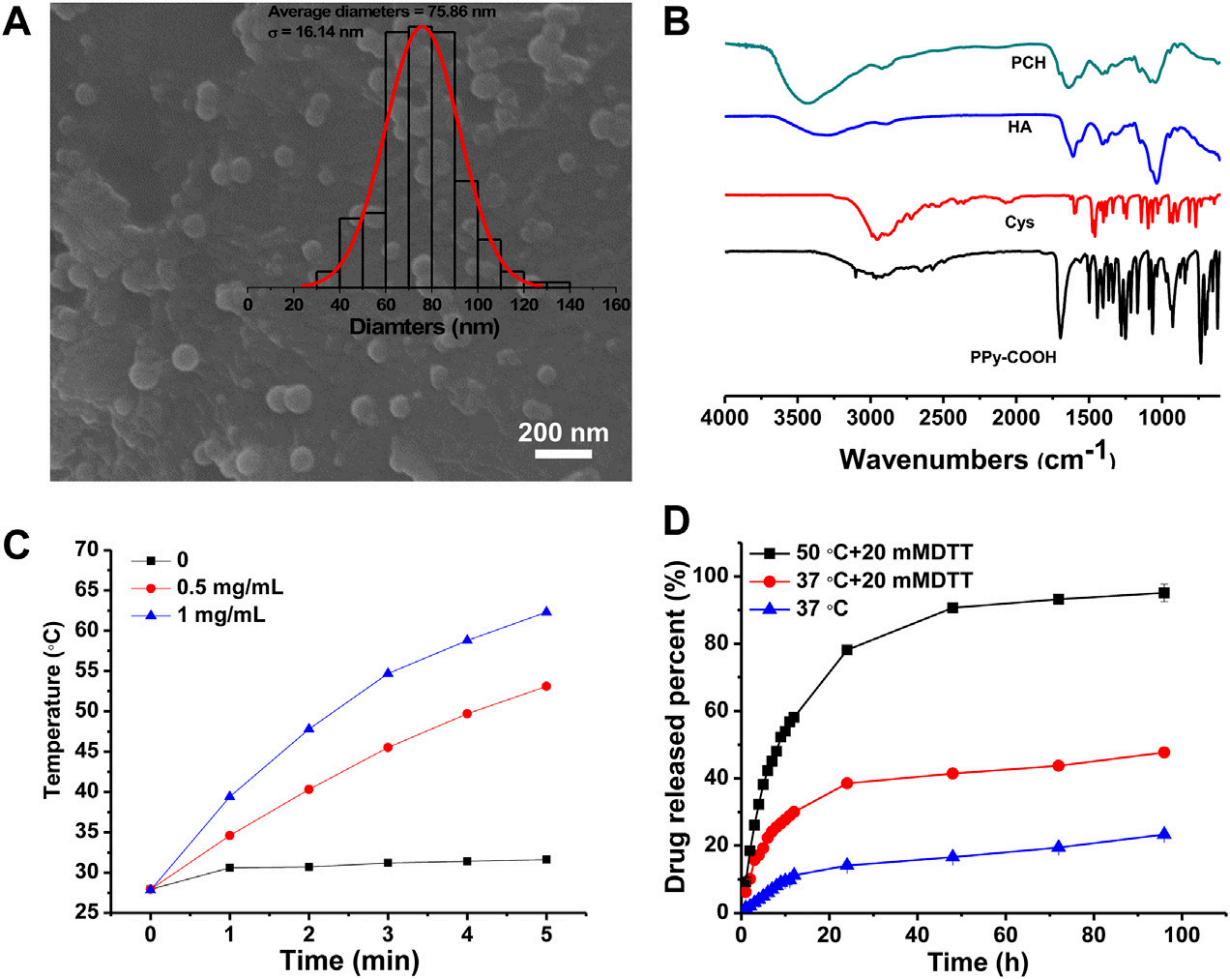
**(A)** SEM micrographs and diameter distribution histograms of PCH nanoparticles. **(B)** FTIR spectrum of PPy-COOH, Cys, HA, and PCH. **(C)** Temperature curves of PCH aqueous solutions (0, 0.5, 1 mg/ml) under laser irradiation of 1,064 nm laser at the power density of 1 W/cm^2^. **(D)** Cumulative release curve of PTX/PCH nanoparticles in different media and temperature.

The synthetic procedure of PCH nanoparticles consisted of three steps. The first step was to prepare PPy-COOH nanoparticles by a one-step aqueous dispersion polymerization of Py and Py-COOH. The second step was the grafting of Cys onto PPy-COOH nanoparticles. The third step was to prepare HA derived PPy nanoparticles. As shown in Figure 1A, PCH nanoparticles were in a spherical shape with a nearly uniform size of 75.86 ± 16.14 nm. This is consistent with previously reported (Guo et al., 2019).

To verify that whether HA molecules were successfully modified onto the surface of PPy nanoparticles, PCH, PPy-COOH and Cys were characterized by FTIR (Figure 1B). PPy-COOH exhibit a peak at 1,690 cm^−1^, attributing to –C=O. Weak peaks at 1,595 cm^−1^ and 1,030–1,230 cm^−1^ are caused by the bending vibration of –NH and the tensile vibration of the Cys C–N bond. In addition, because of the low detection sensitivity limit for the S–S bond strength, no stretching vibration of the disulfide bond (S–S) appears in the FTIR spectra (Rao et al., 2018). For HA, the peaks at about 3,300 cm^−1^ and 2,900 cm^−1^ are owing to the stretching vibrations of O–H/N–H bond and C–H bonds, respectively. The FTIR spectra show peaks at about 1,610 cm^−1^ and 1,405 cm^−1^ and could be ascribed to the stretching vibrations of the C=O and C–O bonds of the –COO– group, while the characteristic peak of C–O–C showed asymmetrical and symmetrical stretching frequencies at 1,145 and 1,040 cm^−1^ (Das et al., 2018). The peaks at 1,560 cm^−1^ are owing to the –NH stretching frequencies in HA. A new absorption peak at 1707 cm^−1^ amide (C=O stretching) appears in the FTIR spectra of the PCH, indicating that the modification caused the formation of amide bonds. All in all, these clearly suggested that HA has been successfully grafted to the surface of PPy nanoparticles.

Previous study has demonstrated that PPy based nanoparticles can absorb the spectrum in the NIR-II range very well (Xu et al., 2022). As shown in Figure 1C, when the solution was irradiated with a NIR-II laser at 1,064 nm, its temperature increased, and the temperature was positively correlated with the PCH concentration. For 1 mg/ml case, the temperature of the solution can reach up to 62.3°C within 5 min of irradiation, confirming the excellent photothermal conversion ability of PPy. Based on Korgel’s method (Xie et al., 2022; Zhang et al., 2022), the photothermal conversion efficiency of the PCH nanoparticle was determined to be 33.48% (Supplementary Figures S1A,B). In the three lasers’ on-off cycles, the maximum temperature of the PCH nanoparticles could remain stable; therefore, the PCH nanoparticle has acceptable photothermal stability (Supplementary Figure S1C).

The release of PTX from PTX/PCH nanoparticles was measured at 37°C or 50°C for 96 h (Figure 1D). 20 mM DTT were used to simulate the high reducing tumor microenvironment. It was found that the cumulative release amount of PTX from PTX/PCH nanoparticles within 96 h was 23.3%, 47.7%, and 95.1% at T = 37°C, T = 37°C/20 mM, T = 50°C/20 mM DTT, respectively. This suggested that the PTX release increased with the temperature rising and in the presence of 20 mM DTT. The increased PTX release rate might be attributed to DTT triggered disulfide bond cleavage and inverse cross-linking of the PCH. Therefore, drug release can be accelerated in an intracellular reducing environment (Zhu et al., 2016), and this controlled drug release is beneficial to the improvement of tumor treatment.

### *In-vitro* cell assay

**FIGURE 2:**
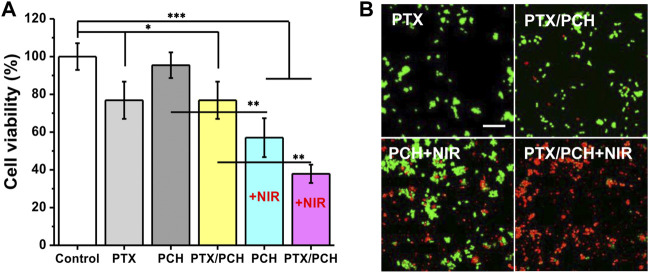
**(A)** *In-vitro* combined tumor photothermal therapy of PCH; **(B)** Morphology of dead/live staining of MDA-MB-231 cells corresponding to **(A)**. Scare bar = 50 μm.

The *in vitro* combined cancer therapy of PTX/PCH nanoparticles was also investigated. As shown in Figure 2A, cells treated with saline remained healthy, while the viability of cells treated with pure PTX and PTX/PCH was significantly reduced (PTX: 76.85%, *p* < 0.05, versus saline; PTX/PCH: 79.25%, *p* < 0.05, versus saline) due to the cell killing effect of PTX. Besides, the proliferation of PCH-treated cells was significantly inhibited after NIR irradiation (57.03%, *p* < 0.001, compared to saline), suggesting the excellent *in vitro* photothermal therapeutic efficiency. In addition, due to the NIR triggered photothermal ablation and PTX induced chromosomal instability (Scribano et al., 2021), cancer cells treated with PTX/PCH NIR were mostly killed with a viability of 37.86% (*p* < 0.001, versus saline), which was significantly lower than that treated with PTX/PCH (*p* < 0.01), pure PTX (*p* < 0.001) and PCH + NIR (*p* < 0.01). Moreover, CCK-8 and dead/live staining results further demonstrated the synergistic chemotherapy and photothermal efficiency of PTX/PCH nanoparticles (Figure 2B).

**FIGURE 3:**
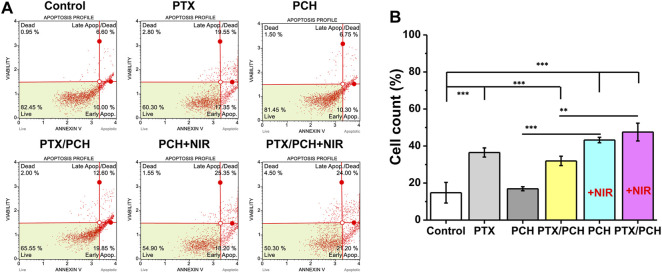
**(A)** Annexin V and Dead Cell analysis method for the apoptosis of MDA-MB-231 cells. **(B)** Apoptosis of MDA-MB-231 cells.

In Annexin V and Dead Cell analysis, the four regions represent dead cells, late apoptosis, early apoptosis, and live cells, respectively, and late + early apoptosis represents total apoptosis. As presented in Figures 3A,B, the total apoptosis rate of cells treated with pure PTX and PTX/PCH was significantly increased (PTX: 36.5%, *p* < 0.001, versus control; PTX/PCH: 31.95%, *p* < 0.001, versus control) suggesting that chemotherapy caused the total apoptosis. PCH + NIR and PTX/PCH was 43.25 ± 1.47% and 47.53 ± 4.80%, respectively, with significant difference from no NIR groups (*p* < 0.001), indicating that NIR led to more apoptosis of cells and the combined treatment group had the best therapeutic effect.

**FIGURE 4:**
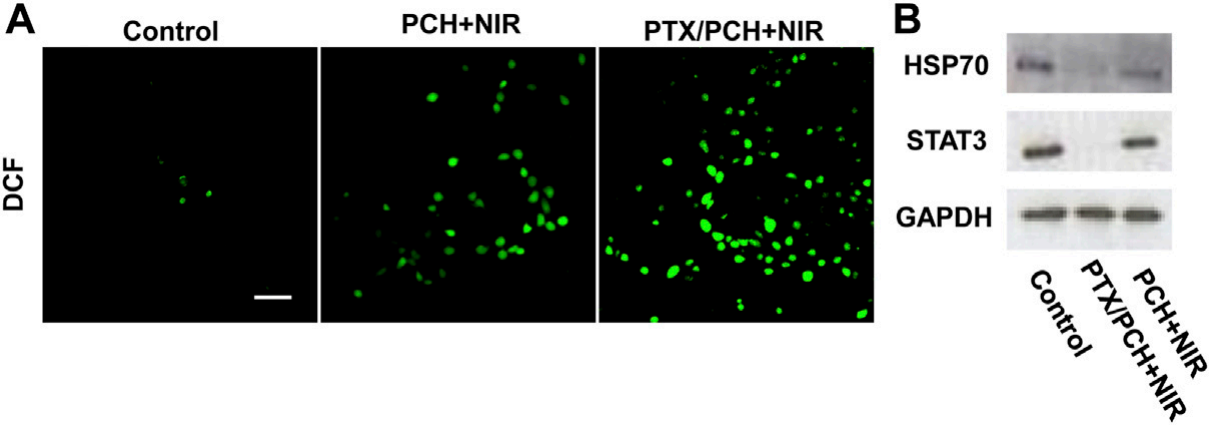
**(A)** ROS production detection after distinct treatments. **(B)** HSP70 and STAT3 expression evaluation. Scare bar = 50 μm.

Reactive oxygen species (ROS) sensitive probe DCFH-DA was applied to investigate the production of ∙OH in cells. The fluorescence intensity increased from PCH + NIR and PTX/PCH + NIR to the control group. The strongest fluorescence was observed in PTX/PCH + NIR group, indicating its strong ability to produce ∙OH and cause cell necrosis (Figure 4A). As shown in Figure 4B, the expression of heat shock protein 70 (HSP70) was inhibited both in the PCH + NIR and PTX/PCH + NIR groups. It was reported that the suppression of HSPs functions can disrupt the cell homeostasis and interfere with the integrity of protein interaction, thereby reducing the cell thermotolerance and improving the efficacy of photothermal therapy (Wang et al., 2016). STAT3 is an oncogene, and the activation and overexpression of STAT3 are relevant with the malignant transformation of cells (Qiao et al., 2016). The expression of STAT3 was suppressed in the PTX/PCH + NIR groups, which will reduce the resistance of tumor cells and promote the therapeutic effect.

**FIGURE 5:**
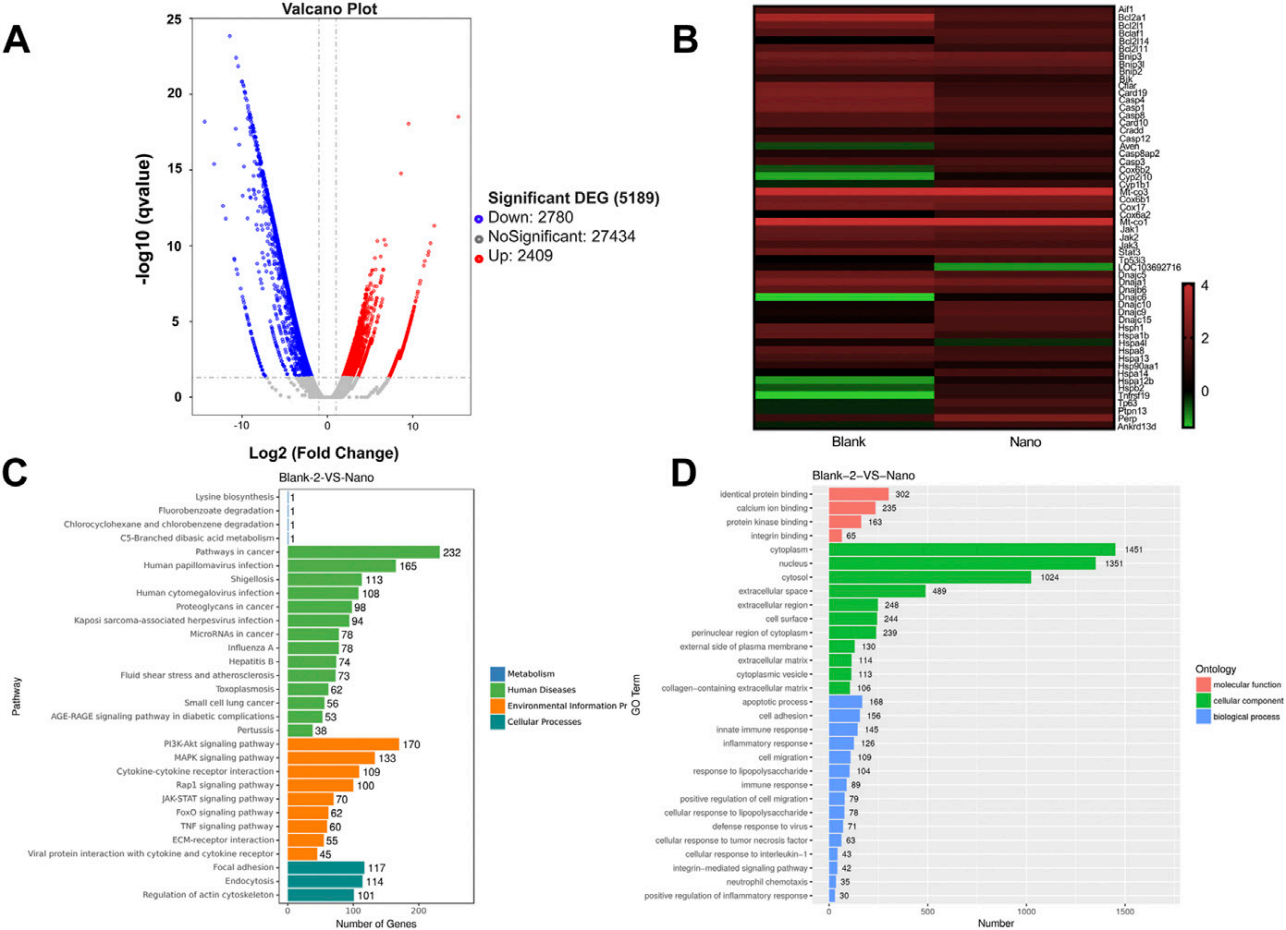
Transcriptomic sequence. **(A)** Volcano map of TCP and PTX/PCH + NIR. **(B)** Heatmap of differentially expressed genes in TCP and PTX/PCH + NIR groups. **(C)** KEGG pathway enrichment. **(D)** GO terms.

Transcriptomic sequencing generated a volcanic map showing 2,780 downregulated and 2,409 upregulated differentially expressed genes in the PTX/PCH + NIR group (Figure 5A). A total of 56 upregulated and downregulated differentially expressed apoptosis related genes were identified. Using the FPKM values of differentially expressed genes under different experimental conditions as expression levels, hierarchical clustering analysis was performed to create a heat map (Figure 5B). Specially, Hsps genes (Hsp40/70 family, Hsp90 family and Hsp100), JAKs genes, Stat3 genes and Bcl-2 genes were downregulated. Bcl-2 is an anti-apoptotic protein and a downstream target gene of START3 (Edmonds et al., 2012). The Kyoto Encyclopedia of Genes and Genomes (KEGG) diagram showed that six pathways were enriched in the PTX/PCH + NIR group, and the higher expression level of the JAK-STAT signaling pathway was also clearly observed (Figure 5C). The gene ontology (GO) analysis showed that enriched GO biological process terms of the PTX/PCH + NIR group were related to associated with apoptotic processes, cell adhesion, and innate immune responses, respectively (Figure 5D).

**FIGURE 6:**
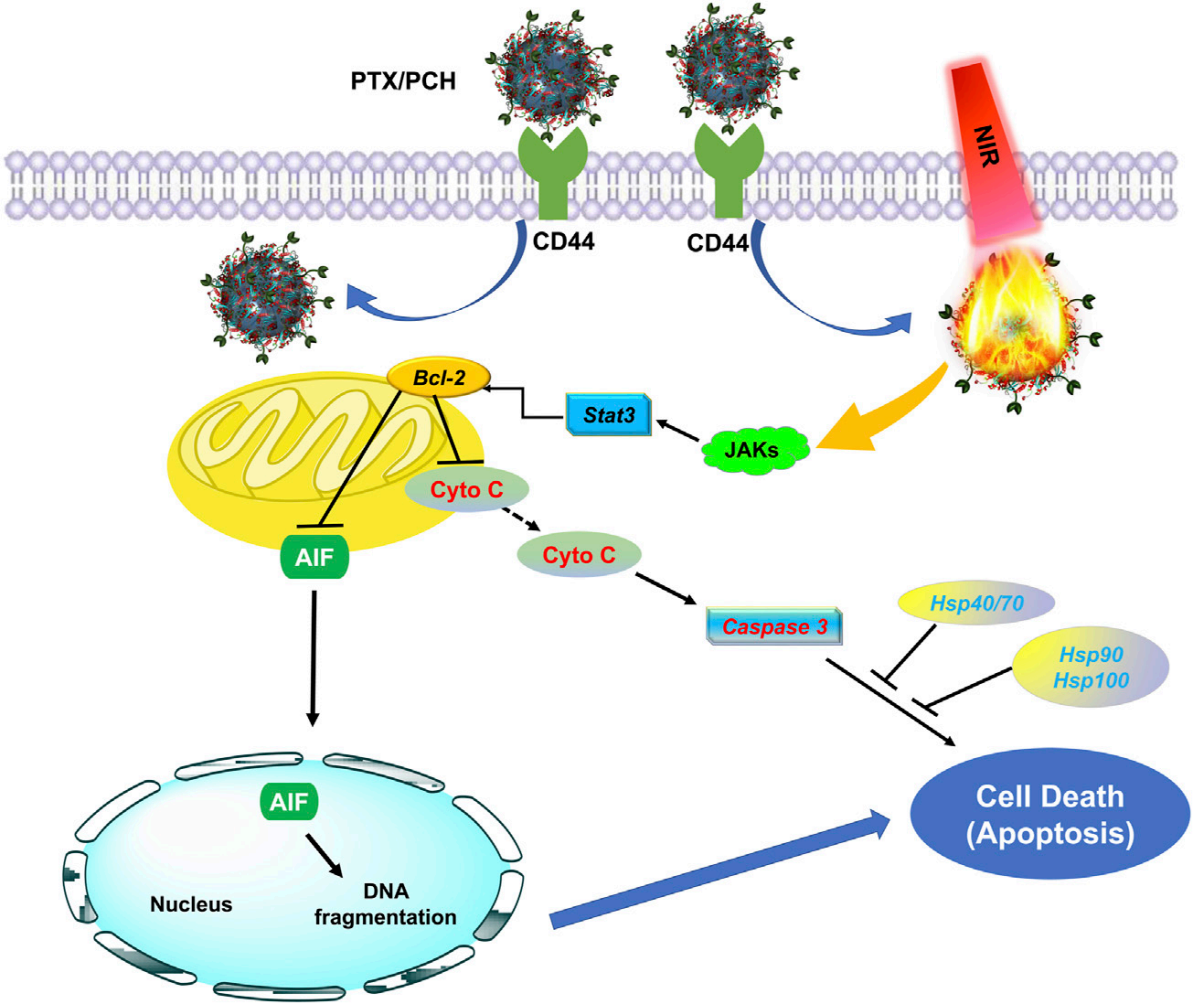
Schematic illustration for the mechanism of PTX/PCH + NIRs induced apoptosis.

Through western blot analysis and transcriptome sequencing, the possible mechanism by which the PTX/PCH + NIRs induced apoptosis of MDA-MB-231 cells is summarized in Figure 6. PTX/PCH + NIRs induced Hsps impaired, heat shock causes the down expression of JAKs, Stat3 and Bcl-2, and the conformational changes of outer membrane of the mitochondria that allows the release of cytochrome c (Cyto C) and apoptosis inducing factor (AIF) (Choi et al., 2011). In turn, apoptotic bodies are formed and apoptosis downstream is carried out by activated Caspase 3. The released AIF enters into the nucleus and causes cell death (Fu et al., 1987).

### *In-vivo* tumor therapy

**FIGURE 7:**
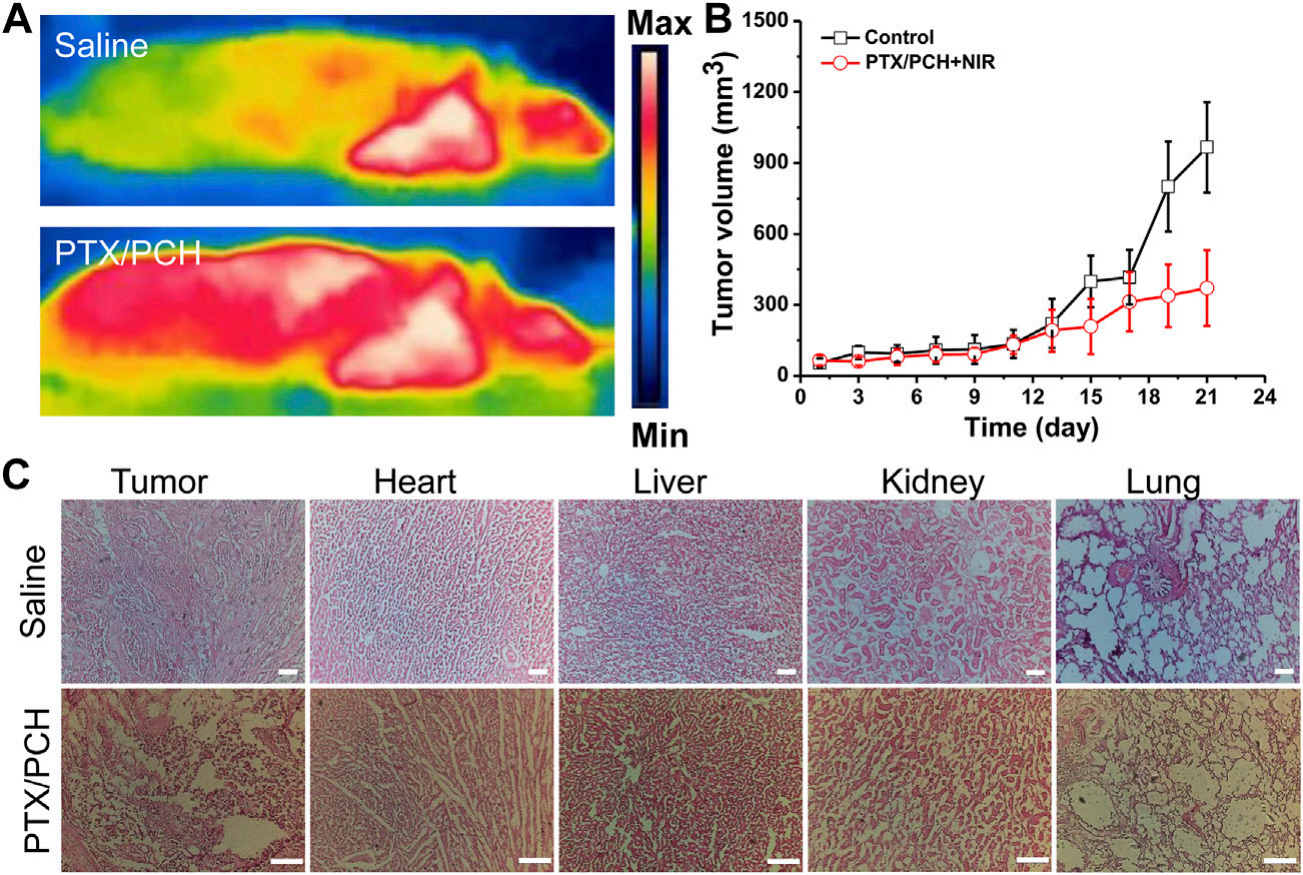
**(A)** Thermal images of the animals treated with saline (NIR) or PTX/PCH (NIR). **(B)** Tumor volume changes. **(C)** The H&E assay indicates themorphological change of the organs (PBS-NIR treated group and PTX/PCH-NIR treated group). Scare bar = 100 μm.

Figure 7A are thermal images of the tumor *in vivo* 2 days after the first administration of NPs and irradiation with NIR for 5 min. Temperatures of the tumors were around 52°C (PTX/PCH + NIRs), and 32°C (NIR plus saline), indicating that a large number of NIR-responsive PTX/PCH NPs may accumulate at the tumor sites after administration. The group that received saline + NIR showed a negligible antitumor efficacy. The group that received PTX/PCH + NIR showed the high antitumor efficacy (Figure 7B) (Feng et al., 2018). Figure 7C shows the histological results. It can be found that the developed PCH administered through local intratumoral delivery into tumor are beneficial because they avoid toxicity to various treatments of surrounding healthy organs (heart, liver, kidney, and lung), they maximize preservation of functional tissue near the tumor. Histological results also revealed that a large number of tumorous cells were mixed with small connective tissue stroma (Figure 7C). Nevertheless, tumor eradication with a luminal structure was observed in the PTX/PCH + NIR treated group, indicating that the PTX/PCH provided a synergistic effect significantly enhanced breast cancer cell death (Figure 7C) (Gao et al., 2014).

## Conclusion

Overall, we reported the synthesis of redox PCH nanoparticles for structural and physicochemical property control for efficient PTT and chemotherapy in breast cancer. The physicochemical and photothermal properties, drug release characteristic, and *in-vitro* and *in-vivo* antitumor therapy efficiency of the devised PCH nanoparticles were studied, and it was found that PTX/PCH nanoparticles induced apoptosis of breast cancer cells through JAK-STAT signaling pathway. The results of this study may provide a new pathway for designing conjugated polymer nanoparticles for breast cancer treatment and other clinical applications.

## Data Availability

The original contributions presented in the study are included in the article/Supplementary Material, further inquiries can be directed to the corresponding authors.
