# Supplementary figures and images for: Redox-responsive hyaluronan-conjugated polypyrrole nanoparticles targeting chemo-photothermal therapy for breast cancer
Source: Front Bioeng Biotechnol. 2022 Oct 24;10:1049437. doi: 10.3389/fbioe.2022.1049437 (PMC9637570; doi:10.3389/fbioe.2022.1049437)

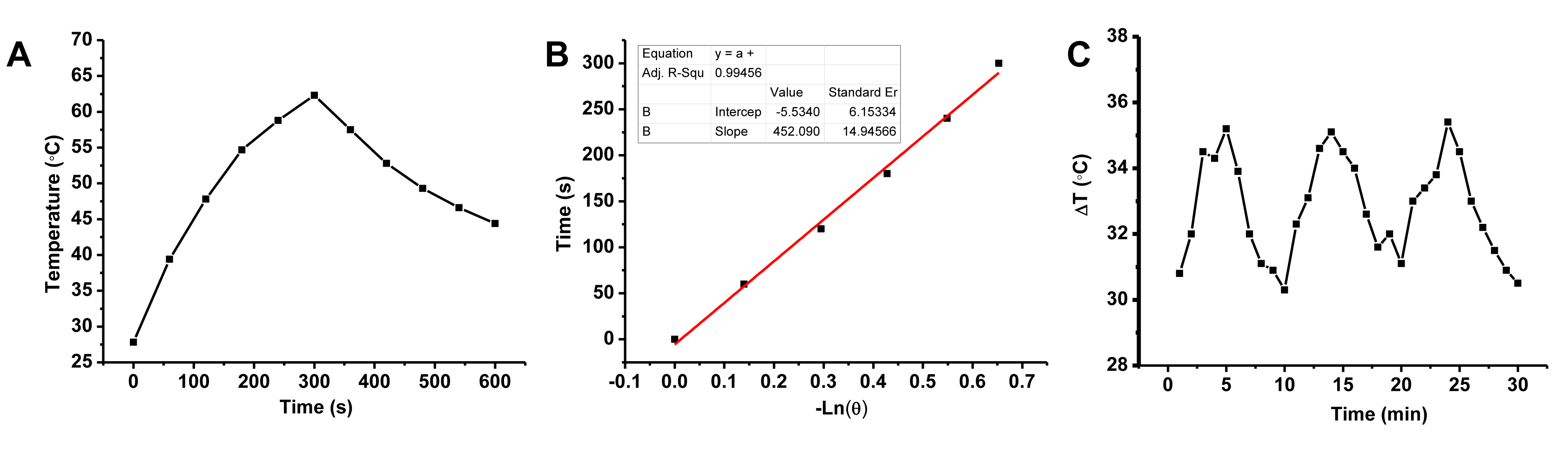

Supplement: Supplementary file 1 [file Image1.TIF]
